# A case report of severe diarrhea due to cytomegalovirus infection after living donor liver transplantation: successful treatment with butyrate-producing bacteria, immunosuppressive adjustments, and anti-cytomegalovirus therapy

**DOI:** 10.20407/fmj.2024-032

**Published:** 2025-08-06

**Authors:** Masanobu Usui, Norimasa Tsuzuki, Miyo Murai, Akihiro Ito, Akihiko Futamura

**Affiliations:** Department of Surgery and Palliative Medicine, Fujita Health University, School of Medicine, Toyoake, Aichi, Japan

**Keywords:** Intractable diarrhea, Liver transplantation, Immunocompromised, Probiotics, Butyrate-producing bacteria

## Abstract

**Purpose:**

In recent years, the benefits of probiotics for perioperative management have been recognized, and butyrate-producing bacteria are attracting attention as new beneficial intestinal bacteria. The present study reports a case of cytomegalovirus (CMV) infection and frequent watery diarrhea caused by immunosuppressants after living donor liver transplantation, wherein administration of butyrate-producing bacteria was considered effective.

**Case:**

The patient was a 75-year-old woman presenting with the chief complaints of weight loss and generalized muscle weakness. History of present illness: Seven years ago, the patient underwent living donor liver transplantation with her daughter as the donor at University Hospital A for liver cancer concurrent with cirrhosis. Following the postoperative outpatient visit, the patient was admitted to a local geriatric care facility because of her advanced age; however, she was urgently rehospitalized at the same university hospital for acute renal failure caused by decreased food intake and diarrhea. Although intensive care saved her life, she was admitted to our hospital for nutritional management and rehabilitation because of considerable weight loss and generalized muscle weakness caused by disuse syndrome resulting from prolonged bed rest. At admission, she was 142 cm tall, weighed 39.5 kg, and had a body mass index 19.6. She was unable to stand up unassisted or transfer to a wheelchair and remained in bed. After admission, central venous nutrition and rehabilitation therapy were initiated. However, on day 10 of admission, she abruptly developed watery diarrhea that occurred 12 times/day, with a blood test showing strong positive results for CMV antigen. Consequently, immunosuppressive drugs were reduced, valganciclovir was administered as an anti-CMV drug, and butyrate-producing bacteria were administered as a probiotic. Subsequently, the watery stools improved, and the frequency of defecation decreased to 4 times/day within 3 weeks after treatment initiation. One month later, she had normal stools twice daily, and CMV antigen results were negative. After her nutritional status improved, the patient was transferred to a different hospital.

**Conclusion:**

We experienced a case in which butyrate-producing bacteria effectively treated a patient with watery diarrhea following liver transplantation.

## Introduction

It is widely known that probiotics play an important role in maintaining and regulating normal intestinal flora in humans.^1^ Moreover, published research and various academic societies in Europe, the United States, and Japan have issued guidelines on the application of probiotics.^2–4^ Recently, among probiotics, butyrate-producing bacteria, which are intestinal bacteria that secrete butyric acid as a short-chain fatty acid, have attracted attention as new beneficial bacteria in the intestines.^5^ Butyric acid is a major energy source for intestinal epithelial cells and has been shown to suppress excessive immune system activity and to have anti-inflammatory effects. While the usefulness of butyrate-producing bacteria has been reported, other lactic acid bacteria and bifidobacteria do not produce butyric acid.

Herein, we report a case of cytomegalovirus (CMV) infection and frequent watery diarrhea caused by immunosuppression after living donor liver transplantation, which was successfully treated with butyrate-producing bacteria.

## Case

A 75-year-old woman

*Chief complaints*: Malnutrition and disuse syndrome

*History of present illness*: Seven years ago, the patient underwent live donor liver transplantation (right lobe graft) with her daughter as the donor for liver cancer concurrent with cirrhosis at University Hospital A. After a long postoperative hospital stay, the patient was treated on an outpatient basis. Thereafter, she was admitted to a local geriatric care facility; however, she developed acute renal failure due to decreased food intake and diarrhea and was urgently admitted to the university hospital, where the transplantation was performed. Moreover, respiratory failure caused by sepsis was observed. Although her life was saved through intensive care, including continuous dialysis ventilator management, and the renal failure and respiratory failure improved, the patient was unable to eat after weaning off long-term ventilation and continuous dialysis. Subsequently, she developed disuse syndrome resulting from prolonged bed rest and was admitted to our department for nutritional management and rehabilitation.

At admission, she was 142 cm tall, weighed 39.5 kg, and had a body mass index (BMI) of 19.6 kg/m^2^. No conjunctival anemia or jaundice was noted. The abdomen was flat and soft, with an inverted T-shaped incision in the upper abdomen and a partial abdominal wall scar hernia. The patient had edema from the bilateral lower legs to the dorsum of the feet. She was extremely emaciated, unable to stand up unassisted or transfer to a wheelchair, and was bedridden. Body composition analyzer (InBody^TM^) measurements revealed that the patient had a muscle mass of 26.3 kg, skeletal muscle mass of 5.9 kg, basal metabolism of 963 kcal, extracellular water content/total body water of 0.433, phase angle of 1.9, and high degree of edema. Furthermore, she was screened as undernourished with a score of 4 on the Mini Nutritional Assessment Short-Form (MNA-SF), and on the Global Leadership Initiative on Malnutrition criteria, she was diagnosed as moderately undernourished because she was >70 years of age as well as had BMI <20, decreased muscle mass, and chronic gastrointestinal absorption disorder.

*Hematologic test findings on admission*: White blood count (WBC) 3,150 /mm^3^, total lymphocyte count 1,880 /mm^3^, hemoglobin (Hb) 9.3 g/dL, total protein (TP) 5.3 g/dL, albumin (Alb) 2.2 g/dL, and total cholesterol 178 mg/dL indicated decreased white blood cells and lymphocyte levels, with anemia and hypoalbuminemia. The patient was moderately malnourished with a controlling nutritional status (CONUT) score of 7 points. C-reactive protein (CRP) 0.3 mg/dL, transthyretin (TTR) 20.1 mg/dL, Mg 1.9 mg/dL, Cu 80 μg/dL, Zn 68 μg/dL, and lactate 11 mg/dL.

*Imaging findings at admission*:

Chest X-ray; no obvious pleural effusion or pneumonia was seen, although the patient could only be supine. Abdominal X-ray found no intestinal obstruction.

Thoracoabdominal CT; no signs of pneumonia or neoplastic lesions were seen. Moreover, no ascites or neoplastic lesions were seen in the right lobe of the transplanted liver.

**Figure 1:**
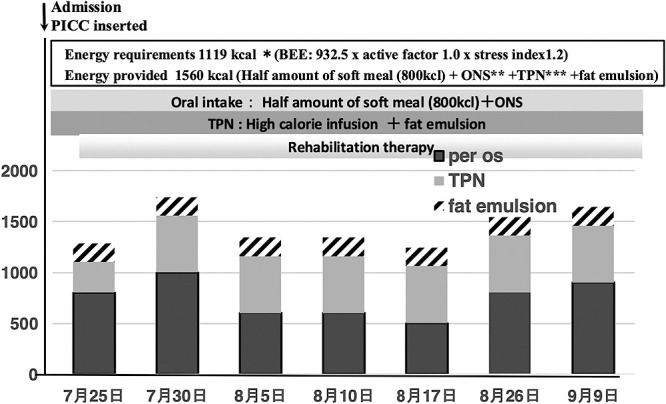
Nutritional management and schedule plan on admission. The patient was scheduled to receive 1560 kcal; oral intake (800 kcal)+oral nutritional supplement (300 kcal/187.5 ml)+total parenteral nutrition (high-calorie infusion+lipid emulsion). She was given total parenteral nutrition consisting of 560 kcal/1000 ml of amino acid, sugar, electrolyte, and trace element preparations and 180 kcal/100 ml of lipid emulsion, totaling approximately 1200–1400 kcal. *BEE: Basal energy expenditure was calculated using the Harris-Benedict formula **ONS: Oral nutritional supplements ***TPN: Total parenteral nutrition

**Figure 2:**
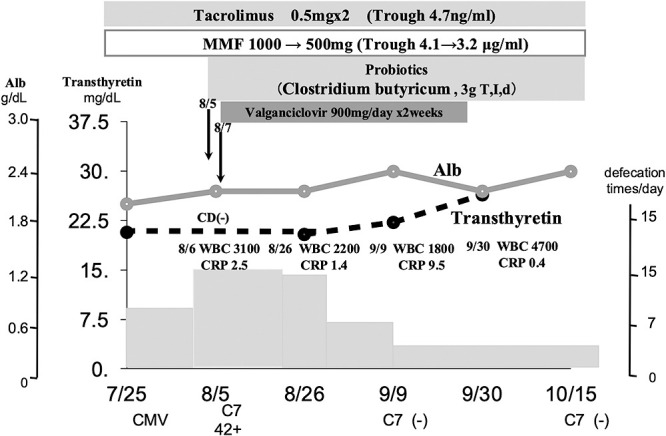
Treatment progress and changes of nutritional indicators after admission. The MMF dose was reduced to 250 mg twice daily. The white blood cell count decreased to 2200/μL, and the CRP level was slightly elevated to 1.4 mg/dL. Valganciclovir was started at 450 mg twice daily as an anti-CMV drug, and a butyrate-producing bacteria preparation was administered at 3 g/day as a probiotic. After about 2 weeks of administration, the number of diarrhea episodes decreased from 12 to 10, and by the third week, the diarrhea was no longer water-soluble at 4, and by the third week, the diarrhea had become normal with 2 normal stools. The white blood cell count decreased to a minimum of 1800/μL, and the CRP level increased to a maximum of 9.5 mg/dL, but by the third week, the white blood cell count had improved to 4100/μL and the CRP level to 1.1 mg/dL. MMF: Mycophenolate Mofetil CMV-C7: Cytomegalovirus-pp65antigen (C7-HRP)

Posthospitalization course: for all patients in our department, the basal energy expenditure is calculated at admission using the Harris–Benedict equation and multiplied by the stress and activity coefficients to establish the total energy expenditure (TEE). As a result, the energy requirement for this case was calculated to be 1119 kcal (TEE 932.5×activity coefficient 1.0×stress coefficient 1.2), and 1560 kcal (half consisting of soft vegetables (800 kcal)+oral nutritional supplements (ONS) (Enoras 300 kcal/187.5 mL)+central venous nutrition (high-calorie infusion formula+fat emulsion) was planned. After admission, a peripheral central venous catheter was placed and central venous nutrition was administered. Because the central venous nutrition management improved general malaise, rehabilitation therapy was initiated (Figure 1). Immediately after admission, 1600 kcal of nutrition was administered as planned; however, the patient’s food intake was irregular, with 50%–70% of the soft vegetable meal ingested and 60% of the ONS ingested. For central intravenous nutrition, 560 kcal/1000 mL consisting of amino acid, sugar, electrolyte, and trace element products with 180 kcal/100 mL of fat emulsion were administered, approximately 1200–1400 kcal in total (Figure 1). On day 10 of admission, the patient suddenly developed watery diarrhea occurring 12 times/day, and simultaneously, CMV antigen CMV-pp65antigen (C7-HRP) was detected abnormally at 42+ via antigenemia blood testing. The stool culture at admission was negative for CD toxin but positive for Candida albicans (1+). Immunosuppressive drugs administered thus far included tacrolimus (tac) at a dose of 0.5 mg twice daily and mycophenolic acid mohethyl (MMF) at a dose of 500 mg twice daily, with blood concentrations of 4.7 ng/mL and 4.1 μg/mL, respectively, at the trough level. TAC was at its lowest level, and there were no problems in blood concentration. However, the MMF concentration was slightly high and considered to oversuppress the immune system; thus, the dose was reduced to 250 mg twice daily while paying attention to liver function. The white blood cell count decreased to 2200/μL, and CRP levels were slightly elevated at 1.4 mg/dL. As a result of reducing the dose of MMF, the trough level decreased to 3.2 μg/mL. Additionally, valganciclovir administration was initiated at a dose of 450 mg twice daily as an anti-CMV drug and 3 g/day of butyrate-producing bacteria as a probiotic. Approximately 2 weeks after administration, the diarrhea frequency decreased from 12 to 10 times per day. By the third week, the diarrhea occurred 4 times per day and was not watery. In 1 month, her stools become normal and were passed twice daily. The white blood cell count decreased to a minimum of 1800/μL, and CRP increased to a maximum of 9.5 mg/dL; however, by week 3, the white blood cell count improved to 4100/μL and CRP to 1.1 mg/dL. Blood sampling results were negative for CMV antigen at 1 month, and stool culture was negative. After the diarrhea improved, as a nutritional indicator, the transthyretin level, which was 20.1 mg/dL on admission, increased from 1 month after the diarrhea subsided to 26.4 mg/dL at 2 months. The patient’s Alb level, 2.2 mg/mL at admission, increased to 3.0 mg/dL at 2 months (Figure 2). The patient’s nutritional condition stabilized, and she was transferred to a convalescent hospital afterward.

## Discussion

Bacteria inhabiting the human intestinal tract form the intestinal microflora and interact with the host’s metabolism to perform various functions, such as regulating nutritional and immune functions.^6^ Among these bacteria, probiotics are defined by Fuller (1989) as “live microbial feed supplement, which beneficially affects the host animal by improving its intestinal microbial balance.”^7^ Recent studies resulted have reports on the therapeutic effects of intestinal flora on various diseases, such as cancer, patients with compromised immunity, and metabolic diseases.^8,9^ Research on the role of intestinal bacteria in developing the host immune system and immune responses has demonstrated that the intestinal flora can stimulate intestinal immunity, activate innate immune responses, trigger physiological inflammation, and, in genetically susceptible individuals, potentially cause persistent or chronic inflammation.^10^ Probiotics are widely used to treat dyslipidemia, intestinal infections, inflammatory bowel syndrome, and cancer because they alter the intestinal flora and have metabolic, immunomodulatory, and antitumor properties.^11^ Particularly, *Clostridium butyricum*, the butyrate-producing bacteria used in this study, is a probiotic that produces short-chain fatty acids, such as butyric, acetic, and propionic acid, which carry out a role in suppressing intestinal inflammation and maintaining normal intestinal function.^12^ Furthermore, it has also been reported that *C. butyricum* can prevent and treat intestinal diseases in animals.^13^ In clinical practice, *C. butyricum* is also used to protect against infection with enterohemorrhagic *Escherichia coli* caused by O157,^14^ and to treat gastrointestinal disorders related to the destruction of gastrointestinal microflora, such as diarrhea and constipation caused by obstructive colitis and pseudomembranous enterocolitis.^15,16^ *C. butyricum* is resistant to gastric acid because of its ability to form spores and repopulate after a decrease in antibiotic concentration following the administration of antibiotics. Considering this ability, butyrate-producing bacteria preparations containing *C. butyricum* as the main ingredient effectively treat and prevent diarrhea caused by antibiotics and during the eradication of *Helicobacter pylori*.^17,18^ In the present case, after 2 weeks of treatment with valganciclovir for CMV infection, the patient’s leukocyte count remained low, and CMV antigen became negative. Valganciclovir was considered effective against CMV infection; however, the CRP levels increased after 2 weeks, suggesting some mixed infection. Because the patient did not have a fever, we did not administer any other antibiotics; however, when antibiotic administration becomes necessary, butyrate-producing bacteria preparations containing *C. butyricum* as the main ingredient may be effective.

Butyrate-producing bacteria is a type of bacteria that ferment and break down dietary fiber delivered to the intestine to produce butyric acid. Butyric acid is a short-chain fatty acid, and butyric acid produced by butyrate-producing bacteria is an important energy source for cells in the large intestine. Other beneficial bacteria in the intestine also metabolize indigestible carbohydrates like oligosaccharides to produce short-chain fatty acids; however, butyrate-producing bacteria are especially important as a major energy source for intestinal epithelial cells, providing approximately 70% of their energy and greatly contributing to the strengthening of the barrier function of the intestinal mucosa.^5^ Particularly, butyric acid is highly bioactivity among short-chain fatty acids, and 95%–99% of the short-chain fatty acids produced in the large intestine are used by intestinal epithelial cells. Moreover, absorbed butyric acid promotes the proliferation of intestinal epithelial cells, act on intestinal peristalsis, and increase intestinal mucosal proliferation, with intestinal peristalsis and the tract itself playing an important role in immunity.^19^

*C. butyricum*, a butyrate-producing bacteria, reduces dextran sulfate-induced enteritis; however, it has been reported that TGF-β and its induction of regulatory T cells (Treg) via proteoglycans are involved in this mechanism.^20^ This suggests that butyrate-producing bacteria play a major role in the prophylactic treatment of patients with compromised immunity in whom increased mucosal permeability is an important etiological factor. Immunosuppressive drugs are necessary for patients for life following organ transplantation, and no obvious bacteria were detected in this case of diarrhea resulting from immunosuppression due to excessive immunosuppressive drugs. Therefore, only butyrate-producing bacteria were administered instead of antimicrobial agents. Butyrate-producing bacteria strongly inhibited the growth of *Vibrio cholerae*, nonagglutinable (NAG) Vibrio, Aeromonas, and Bacillus dysenteriae in mixed cultures, suggesting that TGF-β- and Treg-mediated immune induction may enhance immunity in patients suggested for organ transplant.^21^ It has been reported that using probiotics, especially butyrate-producing bacteria, prevents perioperative infections in patients with living donor liver transplant before surgery.^22,23^ However, even in the long-term following surgery, similar to the current case, patients who underwent organ transplant require immunosuppressants for the rest of their lives, and their immunity often declines after a long period has passed since the surgery, when patients become older, or when they develop cancer due to immunosuppressants. Therefore, it is curial to keep the immune system strong to prevent and treat disease following transplant. In the present case, frequent watery diarrhea was alleviated by reducing the dosage of the immunosuppressant drugs; thereby, reducing immunosuppression and increasing the immunity of the intestinal tract to butyrate-producing bacteria. The patient’s nutritional status, which had been deteriorating due to intractable watery diarrhea, subsequently improved, suggesting that patients could escape from the vicious cycle’s negative spiral.

*Summary*: We experienced a patient with suppressed immunity and frequent watery diarrhea after living donor liver transplantation who responded to butyrate-producing bacteria and nutritional fortification. Therefore, prophylactic administration of butyrate-producing bacteria should be considered for patient with suppressed immunity.

## Supplementary Material

PDF-Japanese

## References

[1] Fuller R. Probiotics in human medicine. Gut 1991; 32: 439–442. 1902810 10.1136/gut.32.4.439 PMC1379087

[2] Bowen JM, Gibson RJ, Coller JK, et al. Systematic review of agents for the management of cancer treatment-related gastrointestinal mucositis and clinical practice guidelines. Support Care Cancer 2019; 27: 4011–4022. 31286233 10.1007/s00520-019-04892-0

[3] Fallone CA, Moss SF, Malfertheiner P. Reconciliation of recent helicobacter pylori treatment guidelines in a time of increasing resistance to antibiotics. Gastroenterology 2019; 157: 44–53. 30998990 10.1053/j.gastro.2019.04.011

[4] Guarner F, Khan AG, Garisch J, et al. World gastroenterology organisation global guidelines: probiotics and prebiotics October 2011. J Clin Gastroenterol 2012; 46: 468–481. 22688142 10.1097/MCG.0b013e3182549092

[5] Roediger WE. The colonic epithelium in ulcerative colitis: an energy-deficiency disease? Lancet 1980; 2: 712–715. 6106826 10.1016/s0140-6736(80)91934-0

[6] Rooks MG, Garrett WS. Gut microbiota, metabolites and host immunity. Nat Rev Immunol 2016; 16: 341–352. 27231050 10.1038/nri.2016.42 PMC5541232

[7] Fuller R. Probiotics in man and animals. J Appl Bacteriol 1989; 66: 365–378. 2666378

[8] Kanazawa H, Nagino M, Kamiya S, Komatsu S, Mayumi T, Takagi K, Asahara T, Nomoto K, Tanaka R, Nimura Y. Synbiotics reduce postoperative infectious complications: a randomized controlled trial in biliary cancer patients undergoing hepatectomy. Langenbecks Arch Surg 2005; 390: 104–113. 15711820 10.1007/s00423-004-0536-1

[9] Usami M, Miyoshi M, Kanbara Y, Aoyama M, Sakaki H, Shuno K, Hirata K, Takahashi M, Ueno K, Tabata S, Asahara T, Nomoto K. Effects of perioperative synbiotic treatment on infectious complications, intestinal integrity, and fecal flora and organic acids in hepatic surgery with or without cirrhosis. JPEN J Parenter Enteral Nutr 2011; 35: 317–328. 21527594 10.1177/0148607110379813

[10] Honda K, Littman DR. The microbiome in infectious disease and inflammation. Annu Rev Immunol 2012; 30: 759–795. 22224764 10.1146/annurev-immunol-020711-074937 PMC4426968

[11] Tanemoto S, Sujino T, Kanai T. Intestinal immune response is regulated by gut microbe. Nihon Rinsho Meneki Gakkai Kaishi 2017; 40: 408–415 (in Japanese). 29367525 10.2177/jsci.40.408

[12] Karasawa K. Iyakuhin toshiteno purobaiothikusu, purebaiothikusu. The Japanese Journal of Clinical Nutrition 2021; 139: 304–309 (in Japanese).

[13] Okamoto T, Sasaki M, Araki K. Rakusankinkeikotoyo niyoru rattoDSSdaicyoen no chiryokoka. Digestion & Absorption 1997;19: 65–68 (in Japanese).

[14] Takahashi M, Taguchi H, Yamaguchi H, Osaki T, Kamiya S. Clostridium butyricum niyoru O-157:H7cyokansyukketsuseidaicyokin eno kansenbogyokouka no kento (Preventive effect of clostridium butyricum on enterohemorrhagic Escherichia coli o157:H7 infection). Prog Med 1997; 17: 1869–1873 (in Japanese).

[15] Yamanaka H. A case of obstructive colitis with sigmoid colon cancer treated preoperatively with oral clostridium butyricum. Online Journal of JSPEN 2023; 5: 155–159 (in Japanese).

[16] Ito I, Hayashi T, Iguchi A, Endo H, Nakao M, Kato S, Nabeshima T, Ogura Y. Effects of administration of clostridium butyricum to patients receiving long-term tube feeding. Nihon Ronen Igakkai Zasshi 1997; 34: 298–304 (in Japanese). 9212685 10.3143/geriatrics.34.298

[17] Kurata S, Taki Y, Inoue K, Miyagawa K. Preventive effect of clostridium butyicum Miyairi (MiyaBM) on antibiotic induced diarrhea in children. Japanese Journal of Pediatrics 1988; 41: 2409–2414 (in Japanese).

[18] Miyagawa N, Takeuchi N, Tanaka M. Helicobacter pylori no jyokinchiryoji ni hasseisuru geri, nanben ni taisuru rakusankinseizai no koka. Japanese Pharmacology & Therapeutics 1999; 27: 1361–1366 (in Japanese).

[19] Yamamoto M, Ohmori H, Takei D, Matsumoto T, Takemoto M, Ikeda M, Sumimoto R, Kobayashi T, Ohdan H. Clostridium butyricum affects nutrition and immunology by modulating gut microbiota. Biosci Microbiota Food Health 2022; 41: 30–36. 35433162 10.12938/bmfh.2021-046 PMC8970657

[20] Kashiwagi I, Yoshimura A. Mechanism of Treg induction by intestinal microbiota. Journal of Clinical and Experimental Medicine 2016; 259: 875–878 (in Japanese).

[21] Kuroiwa T, Kobari K, Iwanaga M. Inhibition of enteropathogens by Clostridium butyricum MIYAIRI 588. The Journal of the Japanese Association for Infectious Diseases 1990; 64: 257–263 (in Japanese). 2193065 10.11150/kansenshogakuzasshi1970.64.257

[22] Mallick S, Kathirvel M, Nair K, Durairaj MS, Varghese CT, Sivasankara Pillai Thankamony Amma B, Balakrishnan D, Gopalakrishnan U, Othiyil Vayoth S, Sudhindran S. A randomized, double-blinded, placebo-controlled trial analyzing the effect of synbiotics on infectious complications following living donor liver transplant-PREPRO trial. J Hepatobiliary Pancreat Sci 2022; 29: 1264–1273. 35583161 10.1002/jhbp.1182

[23] Eguchi S, Takatsuki M, Hidaka M, Soyama A, Ichikawa T, Kanematsu T. Perioperative synbiotic treatment to prevent infectious complications in patients after elective living donor liver transplantation: a prospective randomized study. Am J Surg 2011; 201: 498–502. 20619394 10.1016/j.amjsurg.2010.02.013

