# Supplementary material for: A case report of severe diarrhea due to cytomegalovirus infection after living donor liver transplantation: successful treatment with butyrate-producing bacteria, immunosuppressive adjustments, and anti-cytomegalovirus therapy
Source: Fujita Med J. 2025 Aug 6;11(4):199–202. doi: 10.20407/fmj.2024-032 (PMC12576399; doi:10.20407/fmj.2024-032)
Supplement: Supplementary file 1 — PDF-Japanese [file fmj-11-199-s001.pdf]

Case report

タイトル : CMV 感染による高度下痢に対して、免疫抑制剤の調整、抗 CMV 療法とともに酪酸産生菌の投与が有効であった生体肝移植後の１例

ランニングタイトル : 酪酸産生菌により難治性下痢の改善が見られた肝移植後の１例

臼井正信　都築則正　村井美代　伊藤彰博　二村昭彦

Masanobu Usui, MD, PhD , Norimasa Tsuzuki, MD, PhD , Miyo Murai, MD, PhD , Akihiro Ito, MD, PhD , Akihiko Futamura, PhD

Department of Surgery and Palliative Medicine, Fujita Health University, School of Medicine, Toyoake, Aichi, Japan

Corresponding author: Usui Masanobu, MD, PhD

Department of Surgery and Palliative Medicine, Fujita Health University,

School of Medicine, 1-98 Dengakugakubo, Kutsukakecho, Toyoake, Aichi

470-1192, Japan

Tel: 0562-93-9014

E-mail: masanobu.usui@fujita-hu.ac.jp

Abstract

【目的】近年，周術期管理の上でプロバイオティクスの有用性が認識されるようになってきたが，酪酸産生菌が新たな腸内有益菌として注目を集めつつある． 今回，生体肝移植後の免疫抑制により出現したサイトメガロウィルス(CMV)感染と頻回の水溶性下痢に対し，酪酸産生菌の投与が有効と考えられた１例を経験したので報告する．【症例】75 歳女性．主訴：体重減少と全身筋力低下．【現病歴】肝硬変合併肝癌にて 7 年前に娘をドナーとして A 大学病院にて生体肝移植を施行．術後外来通院の後，高齢のため近くの老健施設に入所となったが，食事摂取量の低下と下痢から急性腎不全となり同大学病院に緊急再入院となった．集中治療により救命したが，体重減少と長期臥床による廃用症候群にて全身筋力低下が著しいため，栄養管理とリハビリ目的で当科入院となった．入院時現症：身長 142cm，体重 39.5kg，BMI 19.6 で自力で立ち上がれず，車いす移乗もできず臥床のままでの生活であった． 入院後中心静脈栄養とリハビリ療法を開始したが 10 日目より突然 12 回/日の頻回の水溶性下痢が出現し，血液検査で CMV 抗原が強陽性で検出された．免疫抑制剤を減量し，抗 CMV 薬としてバルガンシクロビルの投与とプロバイオティクスとして酪酸産生菌製剤の投与を行った．投与開始後 3 週で水様便は改善し，排便回数も 4 回/日となった． 1 か月後には普通便，2 回/日となり CMV 抗原も陰性となった．その後栄養状態は改善し転院となった．【まとめ】肝移植後患者の水溶性下痢に酪酸産生菌が有効であった症例を経験した．
Key word：難治性下痢，肝移植，免疫低下，プロバイオティクス，酪酸産生菌

【はじめに】

プロバイティクスはヒトの正常な腸内常在菌の維持と調節に重要な機能をもっていることは現在広く知られている[1]．様々な研究報告が発表され欧米・日本からプロバイオティクスの有用性が各種学会よりガイドラインとして公表されている[2-4]． 最近，プロバイオティクスの中でも短鎖脂肪酸として酪酸を分泌する腸内細菌, いわゆる酪酸産生菌が新たな腸内有益菌として注目されている[5]．酪酸は腸管上皮細胞の主要なエネルギー源で，過剰な免疫系を抑制し抗炎症効果を持つことが明らかになっており，その有用性が報告されているが，他の乳酸菌やビフィズス菌は酪酸を産生しない.

今回，生体肝移植後の免疫抑制により出現したサイトメガロウィルス感染と頻回の水溶性下痢に対し，酪酸産生菌製剤の投与が有効であった 1 例を報告する.

【症例】75 歳女性主訴：低栄養，廃用症候群

現病歴：肝硬変合併肝癌にて 7 年前に娘をドナーとして A 大学病院にて生体肝移植を施行(右葉グラフト)．術後長期入院の後，外来通院となった．その後高齢のため近くの老健施設に入所したが, 食事摂取量の低下と下痢から急性腎不全となり移植を行った A 大学病院に緊急入院. 敗血症から呼吸不全も認め，持続透析人工呼吸器管理を含めた集中治療により救命し腎不全・呼吸不全は改善したが長期の人工呼吸器からの離脱および持続透析離脱後に食事が摂れず，ほとんどベッド上で過ごす廃用症候群となり，栄養管理とリハビリ目的に当科入院となった.

入院時現症：身長 142cm，体重 39.5kg，body mass index (以下，BMI と略)　19.6 kg/m^2，結膜に貧血・黄疸無く，腹部は平坦軟で上腹部に逆 T 字切開創があり一部に腹壁瘢痕ヘルニアを認めた．両側の下腿から足甲にかけて浮腫を認め，体感は極度の”やせ”で，自力で立ち上がれず，車いす移乗も出来ずベッド上に臥床であった．体成分分析装置（InBody^{TM}) を 用いた測定では, 筋肉量は 26.3kg, 骨格筋量 5.9kg, 基礎代謝 963kcal, 細胞外水分量(ECW)/全身水分量(TBW)は 0.433, 位相角(phase angle;PA) 1.9 と高度の浮腫を認めた．また，Mini Nutritional Assessment Short-Form (MNA-SF) で 4 点の低栄養とスクリーニングされ, Global Leadership Initiative on Malnutrition (GLIM)基準で 70 歳以上, BMI 20 未満と筋肉量の減少, 慢性的な消化管吸収障害をみとめ中等度低栄養と診断された.

入院時血液学的検査所見: WBC 3,150 /mm^3，総リンパ球数 1,880 /mm^3，Hb 9.3 g/dL， TP 5.3 g/dL, Alb 2.2 g/dL , 総コレステロール 178mg/dL と白血球とリンパ球数低下と貧血および低アルブミン血症を認め，CONUT score は 7 点で中等度の栄養不良であった．CRP 0.3 mg/dL，トランスサイレチン (TTR) 20.1 mg/dL, Mg 1.9mg/dL, Cu 80 μg/dL,

Zn 68 μg/dL, 乳酸 11mg/dL であった.

入院時画像所見：

胸部 X-ray:臥位しかとれなかったが, 明らかな胸水や肺炎像は認めなかった. 腹部 X-ray: 腸閉塞などなし.

胸腹部 CT:肺炎像はなく，腫瘍性病変も認めなかった．腹部は右葉の移植肝で腹水や腫瘍性病変を認めなかった.

入院後経過：当科では入院時に全例 Harris-Benedict の 式を用いて基礎代謝量(basal energy expenditure: BEE) の計算を行い，これにストレス係数・活動係数をかけて必要栄養量(total energy expenditure: TEE) の設定を行っている．この結果本症例では必要エネルギー量 1119 kcal (TEE932.5x 活動係数 1.0x ストレス係数 1.2)と算出され, 1560 kcal (軟菜半分量(800kcl)＋経口栄養補助剤（ONS:イノラス®300kcal/187.5ml）+中心静脈栄養(高カロリー輸液製剤＋脂肪乳剤)の投与を計画した．入院後，末梢型中心静脈栄養カテーテルを留置し, 中心静脈栄養を行うとともに中心静脈栄養管理によって全身倦怠感などが改善したため，リハビリ療法を開始した（Figure 1）．入院直後は予定通り 1600kcal の栄養投与ができていたが，その後食事摂取にムラがあり軟菜半分食を 5 割〜7 割接種で ONS も 6 割程度摂取していた．中心静脈栄養としてアミノ酸・糖・電解質製剤・微量元素製剤 560kcal/1000ml と脂肪乳剤 180kcal/100ml で約 1200~1400kcal の投与となっていた（Figure 1）．入院後 10 日目より突然 12 回/日の頻回の水溶性下痢が出現し，同時に血液検査でサイトメガロウィルス(CMV)抗原 Cytomegalovirus-pp65antigen (C7-HRP)がアンチゲネミア法で 42+と異常に検出された．CD トキシンは陰性であったが，入院時の便培養で陰性であったものが *Candida albicans* が 1+で陽性であった．これまで免疫抑制剤はタクリリムス(tac) 0.5mgx2/day とミコフェノール酸モヘチル（MMF）500mgx2/day でありそれぞれの血中濃度はトラフレベルで 4.7ng/mL と 4.1μg/mL であり，tac は最低量で血中濃度も問題なかったが MMF 濃度は若干高いため免疫過剰抑制と判断し，肝機能に注意しながら 250mgx2/day に減量した．白血球は 2200/μL と低下し，CRP は 1.4 mg/dL と軽度上昇していた．MMF を減量した結果トラフ値は 3.2μg/mL と低下した．また抗 CMV 薬としてバルガンシクロビルを 450mgx2/day で投与を開始し，プロバイオティクスとして酪酸産生菌製剤 3g/日の投与を行った. 投与後 2 週間程度で下痢が 12 回が 10 回となり，3 週目には 4 回で水溶性でなくなり，1 か月で 2 回の普通便となった．白血球は最低 1800/μL まで低下し，CRP も最高 9.5 mg/dL まで上昇したが，3 周目に白血球 4100/μL ， CRP 1.1 mg/dL まで改善した．1 か月目の採血で CMV 抗原が陰性となり

便培養も陰性となった．下痢の改善を認めた後，栄養指標として入院時のトランスサイレチン値が 20. 1 mg/dL であったが下痢が治まった 1 か月後より上昇を認め 2 か月後に 26.4 mg/dL となった． これに伴って Alb 値も入院時 2.2mg/mL であったが 2 か月後には 3.0mg/dL まで上昇を認めた（Figure 2）．その後，栄養状態は安定し，療養型病院に転院となった．

【考察】

ヒトの腸管に生息する細菌は腸内細菌叢を形成し，宿主の代謝と相互作用して，栄養機能や免疫機能の制御など，さまざまな機能を果たしている[6)]．その中でプロバイオティクスは， Fuller（1989）により「腸内フローラのバランスを改善することによって宿主の健康に好影響を与える生きた微生物」と定義されている[7)]． 近年の研究により腸内細菌叢は，がんや免疫低下例，代謝性疾患など様々な疾患について治療効果の報告が多く出されている[8,9)]．腸内細菌が宿主の免疫系の発達や免疫応答に おける役割についての研究が進展した結果, 腸内フローラは腸管免疫を刺激し, 自然免疫応答を活性化し, 生理学的炎症を引き起こし, 遺伝学的に病態感受性の強い宿主では持続的な炎症や慢性炎症を惹起する可能性があることが明らかになった[10)]． プロバイオティクスは，腸内細菌叢を変化させ，代謝，免疫調節，抗腫瘍特性があるため，脂質異常症，腸内感染症，炎症性腸症候群，癌の治療に広く使用されている[11)]．特に今回使用した酪酸産生菌であるクロストリジウム・ブチリカムは，酪酸，酢酸，プロピオン酸などの短鎖脂肪酸を生成するプロバイオティクスで，短鎖脂肪酸は，腸の炎症を抑制し，正常な腸機能を維持する役割を果たす[12)] ． また，クロストリジウム・ブチリカムは動物の腸疾患の予防や治療ができることが報告されている[13)]．その他，臨床診療では，クロストリジウム・ブチリカムは O157 による腸管出血性大腸菌に対する感染防御や[14)], 閉塞性大腸炎や偽膜性腸炎などの下痢や便秘など, 消化管微生物叢の破壊に関連する胃腸疾患の治療に使用されている[15,16)]．特にクロストリジウム・ブチリカムは芽胞形成能があるため，胃酸に強く，抗生剤の投与後の抗生剤濃度低下後に再増殖が可能であり，この能力を利用してクロストリジウム・ブチリカムを主成分とする酪酸産生菌製剤は，抗生物質起因性の下痢や *Helicobacter pylori* 除菌時の下痢治療や予防に効果が認められている[17,18)]．今回の症例では, CMV 感染に対する治療としてバルガンシクロビルを 2 週間投与することで, 白血球低下もとどまり CMV 抗原が陰性化した．CMV 感染に関してはバルガンシクロビルの効果が考えられるが，2 週間後の CRP が上昇しており，何らかの混合感染が考

えられた．特に発熱等なかったので他の抗生剤投与は行っていないが，抗生剤投与が必要になった場合もクロストリジウム･ブチリカムを主成分とする酪酸産生菌製剤は有効である可能性がある．

　また，酪酸産生菌は，腸に届いた食物繊維を発酵・分解して「酪酸」を作る細菌の総称で，酪酸は短鎖脂肪酸の一種で，酪酸産生菌が作る酪酸が大腸のエネルギー源として重要である．他の腸内の有益菌でも，オリゴ糖などの難消化性炭水化物を代謝して短鎖脂肪酸を産生するが，酪酸産生菌は特に腸管上皮細胞の主要なエネルギー源として，その70%近くをまかない,腸管粘膜のバリアー機能の強化に大きく関与している[5)]．　特に酪酸は，短鎖脂肪酸の中で最も生理活性が高く，大腸で産生される短鎖脂肪酸の95~99%が腸管上皮細胞で使用される．吸収された酪酸は，腸管上皮細胞の増殖促進作用，腸管の蠕動運動に対する作用，腸管粘膜増殖作用が示され，腸管蠕動や腸管が免疫力に重要な役割を果たしている[19)].

また，酪酸産生菌であるクロストリジウム・ブチリカムはデキストラン硫酸誘導性腸炎を軽減するが，このメカニズムにはプリテオグリカンを介して TGF-βとそれによる制御性 T 細胞(Treg)誘導が関与していることが報告されている[20)]．このことは，粘膜透過性亢進が重要な病因となる免疫低下症例の予防治療に酪酸産生菌が大きな役割を果たすことを予想させる．臓器移植後には，生涯にわたって免疫抑制剤が必要であり，今回の症例も免疫抑制剤の過剰による免疫抑制に伴う下痢に対し, 明かな起因菌が検出されなかったため，抗菌薬の投与は行わず酪酸産生菌製剤のみを投与した．酪酸産生菌は混合培養においてコレラ菌・ナグビブリオ・アエロモナス・赤痢菌の発育を強く抑制しており，TGF-β，Treg を介する免疫誘導が臓器移植患者の免疫力増強になると期待できる[21)]. 生体肝移植に於いて術前よりプロバイオティクス特に酪酸産生菌を使用することで周術期の感染症を予防することが報告されているが[22,23)]，自験例のような術後長期に於いても臓器移植は一生免疫抑制剤が必要であり, 移植後長期が経過して高齢になったり免疫抑制剤により担がん状態になったときに免疫力が落ちていることが多い. したがって, 常に免疫力を高めておくことが移植後の病気の予防や治療にとって重要である. 今回は免疫抑制剤の投与量を減量して免疫抑制を弱め, これに酪酸産生菌による腸管の免疫力を上げたことで頻回の水溶性下痢が軽快した. 難治性水溶性下痢によって落ちていた栄養状態もその後改善していき, 悪循環の負のスパイラルから抜け出すことが出来たと考えられる.

【まとめ】生体肝移植後の免疫抑制患者に起こった頻回の水溶性下痢に対し，酪酸産生菌製剤と栄養強化が有効であった症例を経験した．免疫抑制患者に対しては，予防的に酪酸産生菌製剤の投与を検討すべきと考えられた．

著者にCOIはありません．

参考文献

1) Fuller, R. Probiotics in human medicine. Gut 1991;32:439-42.
2) Bowen JM, Gibson RJ, Coller JK, et al. Systematic review of agents for the management of cancer treatment-related gastrointestinal mucositis and clinical practice guidelines. Support Care Cancer 2019;27:4011-22.
3) Fallone CA, Moss SF, Malfertheiner P. Reconciliation of Recent Helicobacter pylori Treatment Guidelines in a Time of Increasing Resistance to Antibiotics. Gastroenterology 2019;157:44-53.
4) Guarner F, Khan AG, Garisch J, et al. World Gastroenterology Organisation Global Guidelines: probiotics and prebiotics October 2011.J Clin Gastroenterol 2012 ;46:468-81.
5) Roediger WE. The colonic epithelium in ulcerative colitis: an energy-deficiencydisease? .Lancet 1980;2:712-5.
6) Rooks MG, Garrett WS. 2016. Gut microbiota, metabolites and host immunity. Nat Rev Immunol 16: 341–352.
7) Fuller R. Prbiotics in man and animals.　J Appl Bacteriol 1989;66:365-78.
8) Kanazawa H, Nagino M, Kamiya S, Komatsu S, Mayumi T, Takagi K, Asahara T, Nomoto K, Tanaka R, Nimura Y. Synbiotics reduce postoperative infectious complications: a randomized controlled trial in biliary cancer patients undergoing hepatectomy. Langenbecks Arch Surg 2005;390:104-13.
9) Usami M, Miyoshi M, Kanbara Y, Aoyama M, Sakaki H, Shuno K, Hirata K, Takahashi M, Ueno　K, Tabata S, Asahara T, Nomoto K. Effects of perioperative synbiotic treatment on infectious complications, intestinal integrity, and fecal flora and organic acids in hepatic surgery with or without cirrhosis. Journal of parenteral and enteral nutrition 2011;35:317-28.
10) Honda K, Littman DR. The microbiome in infectious disease and inflammation. Annu Rev Immunol 2012;30:759-95.
11) Tanemoto S, Sujino T, Kanai T.cyonaisaikinso to meneki no kakawari Intestinal immune response is regulated by gut microbe. Jpn. J. Clin. Immunol 2017;40 :408-15(in Japanese).
12) Karasawa K.iyakuhin toshiteno purobaiothikusu, purebaiothikusu.rinsyoeiyo 2021;139:304-9(in Japanese).
13) Okamoto T, Sasaki M, Araki K.rakusankinkeikotoyo niyoru rattoDSSdaicyoen no chiryokoka.syokatokyusyu1997;19 ︰65-8(in Japanese).

14) Takahashi M, Taguchi H, Yamaguchi H, Osaki T, Kamiya S. Clostridium butyricum niyoru O-157:H7cyokansyukketsuseidaicyokin eno kansenbogyo no kento preventive effect of clostridium butyricum on enterohemorrhagic Escherichia coli o157:H7 infection ｼ. Prog Med 1997;17:1869-73(in Japanese).
15) Yamanaka H. jyutsuzenrakusankinseizai wo toyo shi heisokusedaicyoen no chiyugo ni taikitekifukukukyokakeccyosetujyo wo seko shita esujyokeccyogan noichire case of obstructive colitis with sigmoid colon cancer treated preoperatively with oral clostridium butyricum. J JSPEN 2023; 5 : 155-9(in Japanese).
16) Ito I, Hayashi T, Iguchi A, Endo H, Nakao M, Kato S, Nabeshima T, Ogura Y. keikaneiyosekocyu no koreikanjyacyonenmakukino ni taisuru rakusankinkendakueki no koka ｼ Effects of Administration of Clostridium butyricum to Patients Receiving Long-Term Tube Feeding .Jpn J Geriat 1997;34:298-304(in Japanese).
17) Kurata S, Taki Y, Inoue K, Miyagawa K. kosebusshitsutoyoji no geri ni taisuru kasseirakusankinseizai ｼ Preventive effect of clostridium butyicum Miyairi(MiyaBM) on antibiotic induced diarrhea in children ｼ. Japanese Journal of Pediatrics 1988;41:2409-14(in Japanese).
18) Miyagawa N,Takeuchi N,Tanaka M. Helicobacter pylori no jyokinchiryoji ni hasseisuru geri,nanben ni taisuru rakusankinseizai no koka. Jpn Pharmacol Ther 1999 ;27: 1361-6(in Japanese).
19) Yamamoto M, Ohmori H, Takei D, Matsumoto T, Takemoto M, Ikeda M, Sumimoto M, Kobayashi T, Ohdan H. Clostridium butyricum affects nutrition and immunology by modulating gut microbiota. Bioscience of Microbiota, Food and Health 2022;41:30–36
20) Kashiwagi I, Yoshimura A. Chonaisaikin niyoru Treg yudono bunshikikou. Igakunoayumi 2016;259:875-878(in Japanese)
21) Kuroiwa T, Kobari K, Iwanaga M.rakusankin niyoru cyokanbyogenkinyokuseisayo Inhibition of Enteropathogens by Clostridium butyricum MIYAIRI 588. kansensyogakuzassi 1990;64:257-63(in Japanese).
22) Shweta M, Manikandan K, Krishnanunni N, Madhu SD, Christi TV, Binoj Sivasankara Pillai Thankamony Amma, Dinesh B, Unnikrishnan G, Sudheer OV, Surendran S. A randomized, double-blinded, placebo-controlled trial analyzing the effect of synbiotics on infectious complications following living donor liver transplant-PREPRO trial. J Hepatobiliary Pancreat Sci 2022;29:1264-73.
23) Eguchi S, Takatsuki M, Hidaka M, Sayama A, Ichikawa T, Kanematsu T. Perioperative synbiotic treatment to prevent infectious complications in patients after elective living

donor liver transplantation: a prospective randomized study. The American journal of surgery 2011;201:498-502.

Legend for figures

Figure 1 Nutritional management and schedule plan on admission. The patient was scheduled to receive 1560 kcal (oral intake (800 kcal) + oral nutritional supplement (ONS:300 kcal/187.5 ml) +total parenteral nutrition (high-calorie infusion + lipid emulsion). She was given total parenteral nutrition consisting of 560 kcal/1000 ml of amino acid, sugar, electrolyte, and trace element preparations and 180 kcal/100 ml of lipid emulsion, totaling approximately 1200 to 1400 kcal.

Figure 2 Treatment progress and changes of nutritional indicators after admission. The MMF dose was reduced to 250mg x 2/day. The white blood cell count decreased to 2200/μL, and the CRP level was slightly elevated to 1.4 mg/dL. Valganciclovir was started at 450mg x 2/day as an anti-CMV drug, and a butyric acid-producing bacteria preparation was administered at 3g/day as a probiotic. After about 2 weeks of administration, the number of diarrhea episodes decreased from 12 to 10, and by the third week, the diarrhea was no longer water-soluble at 4, and by the third week, the diarrhea had become normal with 2 normal stools. The white blood cell count decreased to a minimum of 1800/μL, and the CRP level increased to a maximum of 9.5 mg/dL, but by the third week, the white blood cell count had improved to 4100/μL and the CRP level to 1.1 mg/dL
